# A Multi-Target Motor Imagery Training Using Bimodal EEG-fMRI Neurofeedback: A Pilot Study in Chronic Stroke Patients

**DOI:** 10.3389/fnhum.2020.00037

**Published:** 2020-02-18

**Authors:** Giulia Lioi, Simon Butet, Mathis Fleury, Elise Bannier, Anatole Lécuyer, Isabelle Bonan, Christian Barillot

**Affiliations:** ^1^Univ Rennes, Inria, CNRS, Inserm, IRISA, Rennes, France; ^2^Departement of Physical and Rehabilitation Medicine, Centre Hospitalier Universitaire (CHU) Rennes, Rennes, France; ^3^Departement of Radiology, CHU Rennes, Rennes, France

**Keywords:** neurofeedback, fMRI, EEG, stroke, rehabilitation, multimodal neuroimaging

## Abstract

Traditional rehabilitation techniques present limitations and the majority of patients show poor 1-year post-stroke recovery. Thus, Neurofeedback (NF) or Brain-Computer-Interface applications for stroke rehabilitation purposes are gaining increased attention. Indeed, NF has the potential to enhance volitional control of targeted cortical areas and thus impact on motor function recovery. However, current implementations are limited by temporal, spatial or practical constraints of the specific imaging modality used. In this pilot work and for the first time in literature, we applied bimodal EEG-fMRI NF for upper limb stroke recovery on four stroke-patients with different stroke characteristics and motor impairment severity. We also propose a novel, multi-target training approach that guides the training towards the activation of the ipsilesional primary motor cortex. In addition to fMRI and EEG outcomes, we assess the integrity of the corticospinal tract (CST) with tractography. Preliminary results suggest the feasibility of our approach and show its potential to induce an augmented activation of ipsilesional motor areas, depending on the severity of the stroke deficit. Only the two patients with a preserved CST and subcortical lesions succeeded in upregulating the ipsilesional primary motor cortex and exhibited a functional improvement of upper limb motricity. These findings highlight the importance of taking into account the variability of the stroke patients’ population and enabled to identify inclusion criteria for the design of future clinical studies.

## Introduction

Neurofeedback (NF) consists of training self-regulation of a specific brain function by providing a subject with real-time information about his own brain activity (Sitaram et al., 2017). This is thought to have an impact on the related behavioral or pathological condition. The majority of NF (or brain-computer interfaces, BCIs) approaches have relied solely on one imaging technique, historically on EEG recordings. In applications intended for motor recovery, subjects are usually asked to perform motor imagery since imagining the movement and executing it are considered to involve similar brain areas. This is particularly true if the mental practice is oriented towards a kinesthetic (rather than visual) motor imagery (Solodkin et al., 2004).

EEG offers the advantages of practicability and high time resolution but suffers from limited spatial resolution and access to deep brain structures. This has prompted to explore the implementation of NF approaches with other sensing techniques such as fMRI (Sitaram et al., 2012; Liew et al., 2016), granting high spatial resolution and more precise identification of cortical targets. Thus, fMRI allows for regulation of better circumscribed subcortical structures as compared to other techniques used for NF. However, fMRI is a costly and cumbersome imaging technique, which severely limits scanning time and all the more so training time, while EEG represents a practical and affordable technology, which allows for numerous training sessions and potentially increased efficacy of rehabilitation.

These two imaging techniques appear to be highly complementary from various perspectives. In order to exploit the full potential of both EEG and fMRI for NF training, the work of two groups (including ours: Zotev et al., 2014; Perronnet et al., 2017, 2018) has gone further integrating EEG and fMRI to achieve a more specific and effective regulation during NF. Thus, in these studies, EEG and fMRI signals were simultaneously recorded and fed back by means of visual feedback, allowing the subject to self-regulate jointly the electrical activity of determined scalp regions and the blood-oxygen-level-dependent (BOLD) signal of specifically targeted cortical areas. If Zotev et al. (2014) focused on psychiatric rehabilitation, our group was the first to integrate fMRI and EEG NF to train regulation of motor areas and suggested, in a sample of ten subjects, the added value of combined EEG-fMRI NF (with respect to unimodal EEG or fMRI NF) in terms of brain activation and engagement of the subjects (Perronnet et al., 2017).

Other recent studies have revealed the potential of NF and BCI training for stroke rehabilitation (Cervera et al., 2018), as an alternative or in addition to traditional therapies (Wang et al., 2017), to stimulate neural plasticity and support functional improvement (Grosse-Wentrup et al., 2011). Previous works have also shown that NF can enhance the efficacy of motor imagery training, in terms of eliciting brain patterns relevant to the task (Zich et al., 2015; Bagarinao et al., 2018). These works have implemented unimodal EEG or fMRI NF. In this exploratory study, and for the first time in literature, multisession bimodal EEG-fMRI NF for upper limb motor recovery was tested in four stroke-patients. Given the cost and complexity of performing bimodal sessions and in order to guarantee a sufficient cumulated training time to the patients, they underwent a first bimodal NF session, followed by three unimodal EEG-only NF sessions and a final bimodal NF training. We expected that the patient would develop a strategy during bimodal EEG-fMRI NF training, receiving enhanced information, and then “transfer” it to unimodal EEG sessions, to reach a sufficient training intensity.

Besides, the choice of the cortical target of NF training has a critical impact on the rehabilitation outcome. If ipsilesional primary motor cortex (M1) has been suggested to be the most promising target for an efficient motor recovery (Favre et al., 2014), supplementary motor area (SMA) is easier to engage during motor imagery (Sharma et al., 2006; Mehler et al., 2019) than M1 (Berman et al., 2012; Chiew et al., 2012; Blefari et al., 2015) and may play an important role in restoring motor function in more severely affected patients (Di Pino et al., 2014; Plow et al., 2015). In particular, while several studies have shown robust SMA activation during kinesthetic motor imagery, it is still unclear whether M1 can be consistently activated. Some motor imagery studies reported significant activation (Sharma et al., 2008), however, fMRI NF studies found non-conclusive results at group level (Chiew et al., 2012; Blefari et al., 2015) and one recent study showed deactivation of M1 during kinesthetic motor imagery-based upregulation training of the SMA and M1 (Mehler et al., 2019). M1 involvement may depend on the subject and the nature of performed motor imagery task and there is no evidence that it is consistently activated, at least in short training protocols (Hétu et al., 2013). On these premises, the second important novelty of this study is the definition of an adaptive, multi-target training that more strongly rewarded SMA activation in the first NF training session, yet increased the M1 contribution in later sessions. To this end, we defined an adaptive cortical region of interest (ROI) based on a weighted combination of ipsilesional SMA and M1 activities. We varied the weights across the training sessions in order to guide the patient training towards an improved activation of M1 and neighboring ipsilesional motor areas.

The first aim of this pilot work was to test the feasibility of the multisession EEG-fMRI NF training in stroke patients, in view of designing a randomized controlled trial on chronic stroke patients involving a longer training protocol. Second, we aimed at testing if the multi-target bimodal strategy was implementable and efficient in guiding the patients towards the upregulation of the ipsilesional M1. The relation between NF training efficacy and the integrity of the ipsilesional corticospinal tract (CST) reconstructed from diffusion tensor imaging (DTI) imaging was also investigated. Finally, functional tests were performed in order to evaluate the potential for clinical improvement of multi-target, bimodal NF training.

## Methods

### Participants

Four chronic stroke patients (aged between 54 and 76 years, two females) with mild to severe left hemiparesis (Fugl-Meyer score in the range 14–50) and without major cognitive deficits took part in the study (Table 1). All participants gave their written informed consent and the study was approved by the institutional review board Poitiers III Ouest. This being an exploratory experiment, we referred to a previous clinical trial register (NCT01677091) and asked the patients to sign additional consent before taking part in this pilot. All patients had participated to the registered study, however, they were screened again before enrollment and were all included in this pilot.

**Table 1:** Patients’ demographics, stroke characteristics, and clinical outcomes.

					FMA-UE		
ID	Age	Time since stroke	Stroke type	FA asymmetry	PRE	POST	Change
P01	62	5 years	Hemorrhagic-subcortical	0.105	14	14	0
P02	76	3 years	Ischemic-subcortical	0.0358	19	25	+6 (+31.5%)
P03	68	1 year	Hemorrhagic-subcortical	0.064	50	53	+3 (+6.0%)
P04	51	1 year	Ischemic-cortical	0.05	41	37	−4 (−9.7%)

### NF Training Protocol

**Figure 1:**
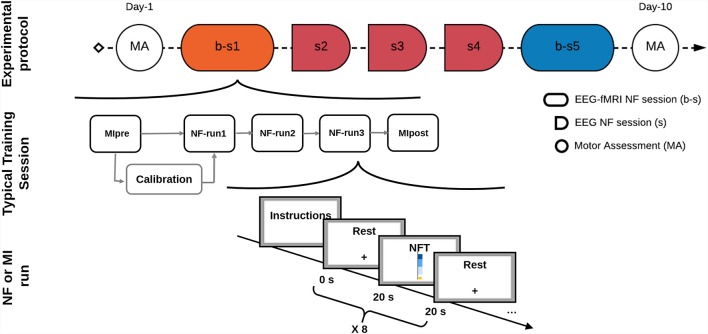
Experimental protocol of multisession Neurofeedback (NF) training procedure. Motor assessment (MA) and NF training sessions timeline is shown in the first row (bimodal EEG-FMRI b-s1 and b-s5 and unimodal EEG s2, s3 and s4). The second row is a schematic of the training protocol that was repeated at each session; finally, the third row shows the time-course of an NF run (block design alternating 20 s rest and 20 s NF training). Abbreviations: b-s, bimodal NF session; s, EEG only NF session; NFT, Neurofeedback training.

The NF training protocol included two bimodal EEG-fMRI NF sessions interleaved with three unimodal EEG NF sessions (Figure 1) and a final motor assessment (MA), within 10 days from inclusion. Patients were informed at the inclusion, verbally and by an explanatory note, about the goals and the timeline of the study. Instructions were repeated before each training session. Instructions for mental imagery oriented the patients towards a technique of kinesthetic motor imagery, without mentioning a specific strategy. For each bimodal NF session, the protocol included a calibration step (motor imagery of hemiplegic hand) and three NF training runs (5 min 20 s each). Each NF run consisted of epochs of rest (20 s) alternated to a period of closed-loop motor imagery training (20 s). Details about the protocol have been previously published by our group (Perronnet et al., 2018). Similarly, the unimodal EEG NF sessions consisted of a calibration period followed by three NF runs with a block-design alternating rest and task during 5 min, with an amount of training time and protocol structure equivalent to bimodal training sessions.

### Data Acquisition and Experimental Setup

**Figure 2:**
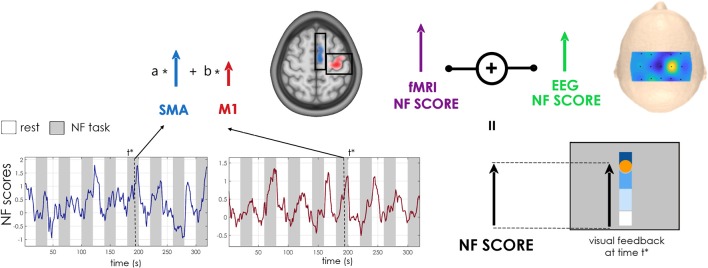
NF calculation schematic. The visual NF at time †* is equal to the average of EEG and fMRI NF scores, updated respectively every 250 ms and 1 s. The fMRI NF score, in turn, is equal to the weighted sum of blood-oxygen-level-dependent (BOLD) activations (contrast NF TASK > REST) in the supplementary motor area (SMA) and primary motor cortex (M1) regions of interest (ROIs) (in blue and red on a normalized anatomical scan, with calibration* a priori* masks in black). The weights assigned to the two contributions M1 and SMA vary from the first training session (*a* = 0.5, *b* = 0.5) to the second (*a* = 0.25, *b* = 0.75). The EEG score was obtained computing the Event Related Desynchronization (ERD) on a combination of electrodes given by Common Spatial Pattern (CSP) or laplacian filter weights.

EEG and fMRI data were simultaneously acquired with a 64-channel MR-compatible EEG solution from Brain Products (Brain Products GmbH, Gilching, Germany) on a 3T Prisma Siemens scanner running VE11C with a 64-channel head coil. EEG data were sampled at 5 kHz with FCz as the reference electrode and AFz as the ground electrode. fMRI acquisitions were performed using echo-planar imaging (EPI) with the following parameters: repetition time (TR)/echo time (TE) = 1,000/23 ms, FOV = 230 × 230 mm^2^, 16 4 mm-slices, voxel size = 2.2 × 2.2 × 4 mm^3^, matrix size = 105 × 105, flip angle = 90°. During rest, the screen displayed a cross and participants were asked to concentrate on the cross and not perform the task. During the task, the screen showed the NF metaphor. The feedback was visual and consisted of a yellow ball moving in a one-dimensional gauge proportionally to the average of the EEG and the fMRI features (Figure 2). As a structural reference for the fMRI analysis, a high-resolution 3D T1 MPRAGE sequence was acquired with the following parameters: TR/TI/TE = 1,900/900/2.26 ms, GRAPPA 2, FOV = 256 × 256 mm^2^ and 176 slabs, voxel size = 1 × 1 × 1 mm^3^, flip angle = 9°. The EEG/fMRI-NF platform in place integrates and synchronizes EEG and fMRI data streams by means of an NF control unit (Mano et al., 2017). EEG data were pre-processed on-line with BrainVision Recview software 2.1.2 (Brain Products GmbH, Gilching, Germany) for gradient and BCG artifact correction (Allen et al., 2000) and sent to the NF control unit for further processing. fMRI data were pre-processed online for motion correction and EEG and fMRI NF features were then computed in the NF control unit using a custom made script developed in Matlab 2017 and SPM8 (The Math-Works, Inc., Natick, MA, USA) and translated as a visual feedback with Psychtoolbox 3^1^.

### Calibration

At the beginning of each experimental session, a motor imagery task without NF was performed to calibrate both fMRI and EEG signals. Immediately at the end of this motor imagery run, EEG and fMRI data were pre-processed and analyzed to estimate subject-specific EEG and fMRI NF calibration features.

For the EEG calibration, only 18 channels located over the motor regions were selected for further analysis. The power in the 8–30 Hz frequency band was computed and a Common Spatial Pattern (CSP; Ramoser et al., 2000) filter was estimated. In cases where the CSP filter did not look physiologically plausible [visual inspection to check if the Event-Related Desynchronization (ERD) computed from filtered data was correlated with the task (Pfurtscheller and Lopes da Silva, 1999)], we used a laplacian filter over the ipsilesional motor electrode C4 (more details in Perronnet et al., 2018). An ERD feature was then computed from filtered data and the threshold for the EEG NF was set equal to the ERD value reached at least 30% of the time. The threshold was computed with the rationale of adapting the NF difficulty to individual performances for each session and make the training engaging.

For the fMRI calibration and the definition of ROIs, data of the motor imagery session were pre-processed for motion correction, slice-time correction, spatial realignment with the structural scan and spatial smoothing (6 mm FHWM Gaussian kernel). A first-level general linear model analysis was then performed. The corresponding activation map was used to define two ROIs around the maximum of activation in the ipsilesional M1 and SMA respectively. To this end two large apriori masks were defined (see Figure 2) and the respective ROIs identified taking a box of 9 × 9 × 3 voxels (20 × 20 × 12 mm^3^) centered around the peak of activation inside the apriori masks. A weighted sum of the BOLD activity in the two ROIs was then used to compute the fMRI NF (Figure 2). Also for the fMRI NF, a threshold was set by estimating the value reached 30% of the time during the calibration session.

### NF Online Calculation

Calibration parameters were estimated before the first NF training run for each training session in order to properly compute the NF features. NF calculation, which has been described in detail elsewhere (Perronnet et al., 2018), was performed on the two synchronized data streams (EEG and fMRI) in the NF control unit. EEG data were firstly filtered with the subject-specific spatial filter selected during the calibration phase. The band power (BP) in the 8–30 Hz band was then computed and normalized with respect to the power in the last 5 s of the previous rest block (prev-rest) with the following event-related desynchronization (ERD; Pfurtscheller and Lopes da Silva, 1999) formula:

(1) $$EEG_{\text{nf}}(t)=(BP_{\text{prev}-\text{rest}}-BP(t))/BP_{\text{prev}-\text{rest}}$$

EEG-NF values were smoothed, divided by the calibration threshold and normalized between 0 and 1 in order to return only positive values to the subject. The EEG feature was eventually translated as visual feedback (position along the gauge) every 250 ms.

The fMRI NF feature (equation 2) was calculated as the difference between percentage signal change in the two ROIs (SMA and M1) and a large deep background region (slice 3 out of 16) whose activity is not correlated with the NF task, in order to reduce the impact of global signal changes (i.e., breathing, heart rate changes and head movements; Thibault et al., 2018).

(2) $$fMRI_{\text{nf}}(t)=a\frac{B_{\text{sma}}(t)}{B_{\text{sma}}(prev-rest)}+b\frac{B_{\text{m1}}(t)}{B_{\text{m1}}(prev-rest)}-\frac{B_{\text{bg}}(t)}{B_{\text{bg}}(prev-rest)}$$

B_sma_ is the average bold signal in the SMA ROI, B_m1_ in the M1 ROI and B_bg_ in the background slice. During the 1st week, the same weight was given to both ROIs (*a* = *b* = 0.5) while in the second session a higher weight was assigned to the BOLD signal of the M1 ROI (*a* = 0.25, *b* = 0.75), in order to guide the training towards upregulation of the ipsilesional motor cortex. The fMRI feature was smoothed over the previous three volumes, divided by the individual threshold and eventually translated as visual feedback every repetition time (1 s). The total position of the ball on the gauge was at every instant equal to the mean between the EEG and fMRI NF features (Figure 2).

## Unimodal EEG-NF

We used the Mensia Modulo (MENSIA TECHNOLOGIES^2^) hardware solution to perform the unimodal EEG-NF sessions. Mensia Modulo is equipped with an 8-channel EEG cap that can be rapidly set up and is designed for a high number of training sessions. The patient received the visual feedback metaphor on a computer screen. The gauge was accompanied by a puzzle game that was completed less or more rapidly depending on the feedback score. Pre-processing included filtering and eye blink artifacts removal (details about the data pre-processing pipeline can be found in the patent US 2017/0311832). An analysis based on the covariance matrix of the ipsilesional motor channels EEG signals was then applied and the ERD NF score was extracted based on the Riemannian distance (Förstner and Moonen, 1999) between motor imagery task and resting blocks.

### Evaluation of Outcome Measures

#### Clinical Outcomes

Before and after the NF training protocol upper limb motor function was assessed by a certified physiotherapist by means of the Fugl-Meyer upper extremity test (FMA-UE; Fugl-Meyer et al., 1975), which evaluates motor activity skills and selectivity of the movement. The FMA-UE score ranges between 0 and 66, with scores lower than 20 indicating severe deficit and scores higher than 48 associated with mild motor impairments (Woodbury et al., 2013).

Subjective ratings on motivation and satisfaction with NF protocol features (i.e., number and length of training sessions, NF metaphor) were evaluated with qualitative questionnaires, based on a 5-point Likert scale (Likert, 1932). Additional comments mainly regarding the motor imagery strategy were noted too.

#### Assessment of Corticospinal Tract Integrity

The integrity of the CST is a well-established predictor of the potential for motor improvement (Stinear et al., 2007). In order to assess the asymmetry between the ipsilesional and contralesional CST, diffusion imaging (TR/TE = 11,000/99 ms, FOV 256 × 256 mm^2^, 60 slices, matrix 128 × 128, voxel size, 2 × 2 × 2 mm^3^, 30 directions, *b* = 1,000 s/mm^2^) was performed at inclusion. The diffusion tensor model was estimated and the fractional anisotropy (FA) calculated. The CST was then reconstructed using the method of Jong et al. (2005) using the software medInria^3^: After estimating the FA maps, two regions of interest were segmented to isolate the CST: the posterior limb of the upper internal capsule and a the CST at the lower pons. FA asymmetry between the affected and unaffected CST was then calculated. FA is a measure of white matter fibers integrity and a disruption of the structural fibers is associated with an FA decrease. An index of FA asymmetry = (FA_contralesional_ − FA_ipsilesional_)/(FA_contralesional_ + FA_ipsilesional_) gives therefore important indications about the structural deficit in the ipsilesional CST. Such an index ranges between −1.0 and +1.0, where positive values indicate reduced FA in the affected CST, and a value of 0 indicates symmetrical FA, i.e. preserved ipsilesional CST. In particular it has been shown that a FA asymmetry index value greater than 0.15 is a “point of no return,” beyond which limited capacity for recovery is expected (Stinear et al., 2007, 2012).

#### fMRI and EEG Outcomes

For each patient, in order to evaluate the effect of NF training on upregulation of BOLD activity, we assessed the difference between SMA and M1 NF scores in session b-s1 and b-s5 by means of a Wilcoxon test across NF runs. We also computed equivalent NF scores for a “neutral” ROI, whose activity is not expected to be upregulated after the motor NF training. Using the Montreal Neurological Institute (MNI) atlas, a 9 × 9 × 3-voxel ROI around the peak of activation in the right medial superior frontal cortex was identified: NF scores were then computed applying the same algorithm as for SMA and M1.

A whole-brain analysis was also performed to characterize cortical areas engaged during NF and describe the reorganization of motor maps at the end of the protocol. Pre-processing (slice-time and motion correction, co-registration to the 3D T1, followed by spatial smoothing with a 6 mm FWHM Gaussian kernel and normalization to MNI template) and a first-level general linear model analysis were performed. The activations maps were voxel-wise Family-Wise error (FWE) corrected (*p* < 0.05).

Similarly, for the EEG analysis data were first pre-processed offline with a semi-automatic artifact rejection procedure implemented in Brain Products Analyzer (version 2.1.1.327) and fieldtrip-20191231^4^; data were then filtered between 8 and 30 Hz using a Butterworth zero-phase filter (48 dB slope). For each subject, mean NF scores per session and the ERD scalp distributions over motor channels were computed for both the bimodal and unimodal training sessions.

For additional details on the methodology of acquisition, processing and analysis of data, including toolbox and software used see Mano et al. (2017); Perronnet et al. (2017, 2018). Data and materials are available upon request to interested researchers.

## Results

**Figure 3:**
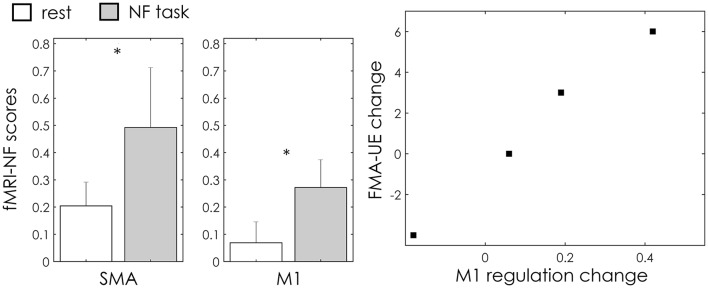
Group results. **(A)** fMRI NF scores values (mean ± standard error across subjects and NF runs) with relative statistics; *indicates statistically significant difference (*p* < 0.01) between rest and NF task as assessed with a Wilcoxon test across subjects. **(B)** Scatter plot relating change in the clinical outcome (FMA-UE score) and ipsilesional M1 BOLD regulation for the four patients.

Overall, in all the patients motor imagery elicited activation, with respect to rest, in the SMA (*p* = 0.004, Wilcoxon test) and M1 (*p* = 0.006, Wilcoxon test) areas (Figure 3A). Two over four patients showed a significant increase in ipsilesional M1 activation (NF score) in the second training session as compared to the first one (Table 2). Interestingly these two also improved their clinical FMA-UE score (Figure 3B). The fourth patient, on the other hand, significantly increased its activation in the SMA area, but decreased it in the ipsilesional M1, and showed a decrease in FMA-UE score. Changes in regulation of the “neutral” ROI in the frontal superior cortex were not significant or in contrast with the upregulation of the motor areas.

**Table 2:** Multi-target fMRI control: individual results of blood-oxygen-level-dependent Neurofeedback (BOLD NF) scores (normalized percent signal change from baseline-rest).

ID	Ipsilesional M1 regulation			SMA regulation			Frontal Sup regulation
	s1	s5	Change	s1	s5	Change	Change
P01	0.10 + 0.25	0.16 + 0.31	+0.06	0.51 + 0.48	0.20 + 0.37	**−0.30**	−0.14
P02	0.23 + 0.37	0.65 + 0.42	**+0.42**	0.19 + 0.27	0.42 + 0.44	**+0.23**	**−0.18**
P03	0.18 + 0.27	0.37 + 0.36	**+0.19**	0.27 + 0.38	0.28 + 0.35	+0.01	−0.05
P04	0.30 + 0.33	0.23 + 0.33	−0.08	0.13 + 0.23	0.21 + 0.25	+0.07	0.05

*Results in bold are significant (p < 0.01) as assessed with the Wilcoxon test across blocks of the same training session. The last column shows results from a “neutral” region: the right Frontal Superior Medial cortex, whose NF scores change between b-s1 and b-s5 was calculated with the same algorithm as for the M1 and SMA*.

Qualitative questionnaire results indicated that generally patients were highly motivated to engage in NF training and very interested by this type of reeducation, which they found complementary with traditional rehabilitation therapies. They were also satisfied with the visual feedback appearance and how it translated their motor imagery effort. Concerning the strategies employed by the patients to control the ball movement, they all used motor imagery of the affected limb. While some of them evoked simple and repetitive tasks (i.e., P01: thought of opening and closing the hand, P04: holding something with the hand), some others engaged in the imagery of a more complex task (P02: imagined hair combing, P03: ironing). Interestingly enough, these more elaborated strategies were also the most effective.

### Individual Results

#### P01

**Figure 4:**
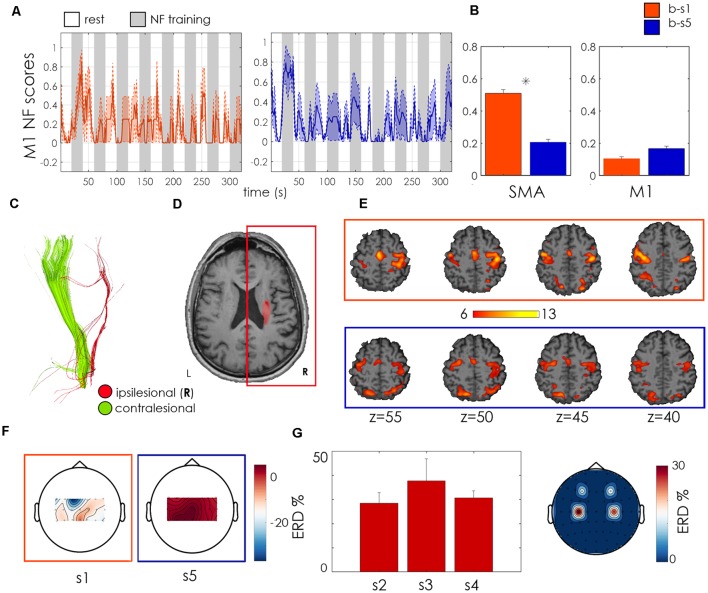
Patient P01 outcome measures. **(A)** M1 regulation during NF training: Normalized NF scores as showed to the patient (mean + standard error across NF sessions—NF1, NF2, NF3- for sessions b-s1 (orange) and b-s5 (blue). Resting blocks are indicated in white, NF training blocks in gray. **(B)** Bar plots of mean normalized NF scores in SMA (left bar plot) and M1 (right) with relative standard error and statistics for b-s1 and b-s5. *Indicates statistically significant difference (*p* < 0.01) between b-s1 and b-s5 as assessed with a Wilcoxon test across blocks of the same training session. **(C)** Corticospinal tract (CST) reconstruction from diffusion MRI imaging. Ipsilesional CST is represented in red and contralesional CST in green. **(D)** Manual Lesion Segmentation (in red) on an anatomical scan. **(E)** Individual contrast activation maps (NF TASK > REST, voxel-wise Family-Wise error (FWE) corrected, *p* < 0.05) during NF training in session b-s1 (orange) and b-s5 (blue). **(F)** Scalp plots of mean EEG ERD (across NF runs) in b-s1 (left) and b-s5 (right; bimodal EEG-fMRI sessions). **(G)** Unimodal EEG-NF outcomes: mean and standard error ERD estimated from the ipsilesional motor electrode (C4) for the three unimodal EEG-NF training sessions (left) with topoplot of the mean ERD values over motor electrodes (right). Results shown in panels **(F,G)** were obtained offline. For each motor channel (18 for the bimodal sessions, five for the unimodal EEG-NF runs) ERD was computed as the normalized difference in the 8–30 Hz band power (BP) between the rest block and the following training block. The mean ERD value for each channel is displayed in scalp plots representing “ERD activation maps.” For panel **(G)**, in order to have a synthetic view of the ERD across the three unimodal sessions, only the ERD from channel C4, the electrode corresponding to the ipsilesional M1, was shown.

The patient was a 62 years old male with right ischemic capsulo-lenticular lesion (Figure 4D) with important loss of ipsilesional CST integrity (Figure 4C) at the level of the posterior limb of the internal capsule (FA asymmetry index = 0,105). Time since stroke was 5 years and the initial FMA-UE score was 14. This patient increased his NF score in the ipsilesional M1 in the second session as compared to the first one, but its activation was relatively weak (Figures 4A,B). The whole-brain analysis revealed a bilateral activation of M1 and SMA during the NF training (Figure 4E). Its EEG activation was bilateral too, and he showed a positive, relatively strong ERD across the three unimodal training sessions (Figure 4G). EEG acquired during the bimodal NF sessions was particularly noisy in session b-s1, and the ERD calculated over ipsilesional electrodes (C2, C4, C6) was negative. In the second session b-s5 the average ERD was positive but relatively small (Figure 4F). No changes in the FMA-UE score were observed at the end of the training.

#### P02

**Figure 5:**
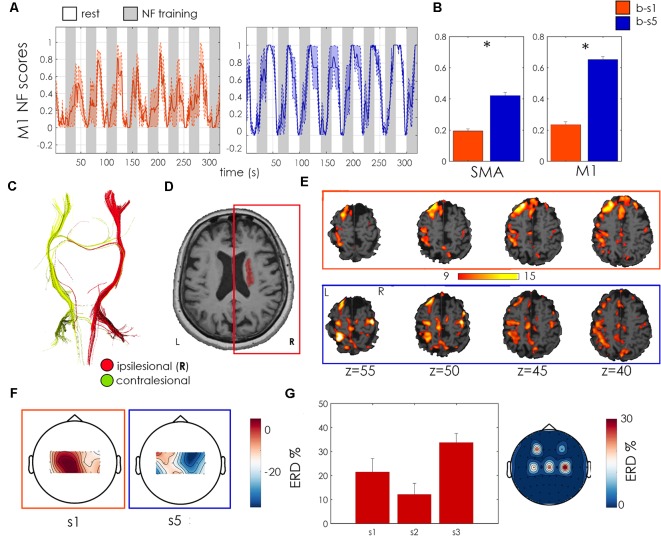
Patient 02 outcome measures. Legend as for Figure 4.

Patient 2 was a 76 years old woman with a right ischemic capsulo-lenticular lesion with a high ipsilesional CST integrity (FA asymmetry index = 0.04; Figures 5C,D). Time since stroke was 3 years and initial FMA-UE score was of 19. Even if showing a vast bilateral activation during motor imagery (Figure 5E), the patient significantly improved volitional control of ipsilesional M1 at the end of the training (*p* < 0.001, Wilcoxon test across 24 training blocks, Figures 5A,B) exhibiting very effective and robust NF trends in the second bimodal training session b-s5. ERD maps of unimodal EEG NF indicate a positive and bilateral activation of the motor channels and ipsilesional ERD was positive for all the unimodal sessions (Figures 5F,G). These functional changes were accompanied by a clinically relevant (Page et al., 2012) increase in the FMA-UE score from 19 to 25 (Table 2).

#### P03

**Figure 6:**
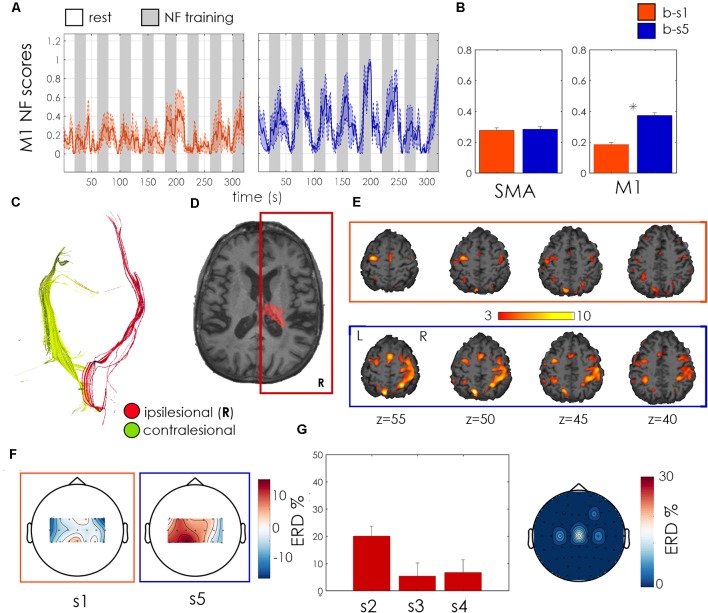
Patient 03 outcome measures. Legend as for Figure 4.

Patient 3 was a 68 years old woman with a right hemorrhagic subcortical lesion (Figure 6D) and a mild hemiparesis (FMA-UE score 50). Time since stroke was 1 year and the symmetry of CST quite well preserved (FA asymmetry index = 0.06, Figure 6C). The patient showed a strong SMA activation, which increased along with the two sessions, as revealed by the BOLD analysis. She significantly increased the activation of the ipsilesional M1 at the end of the training (*p* < 0.001, Wilcoxon test, Figures 6A,B) and exhibited a larger involvement of the ipsilesional motor and premotor areas, with respect to contralesional ones, during the second NF training, as revealed by BOLD activation maps in Figure 6E. The fMRI NF scores during the second session exhibited higher regularity and amplitude, with respect to the first one. During both unimodal and bimodal training, EEG activation was higher for midline motor electrodes (Figures 6E–G). These functional changes were associated with an increase of 3 points of the FMA-UE score (Table 2).

#### P04

**Figure 7:**
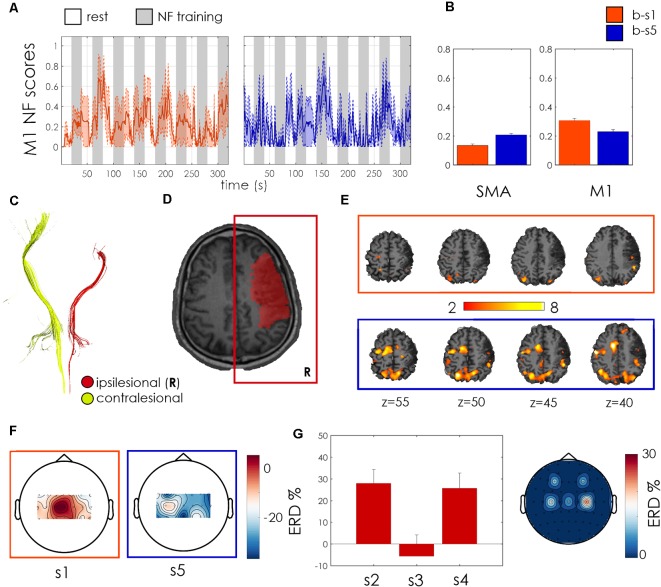
Patient 04 outcome measures. Legend as for Figure 4.

Patient 4 was a 51 years old male affected by a right ischemic-cortical stroke (Figure 7D), which occurred 12 months before the onset of the study. His initial FMA-UE score was 41 and he showed high integrity of the ipsilesional CST, with an FA asymmetry index of 0.05. This patient showed a relatively weak BOLD motor activation during NF training, in particular in the ipsilesional motor cortex (Figure 7E). He exhibited an increase in SMA activation in the final session associated however with a down-regulation of ipsilesional M1 activation, in contradiction with the designed training strategy (Figures 7A,B). This was associated with a negative ipsilesional EEG ERD during the second bimodal NF training session (Figure 7F), and to scarce performances during unimodal EEG sessions (in one session the average ERD was negative and average scalp plot revealed an ERD smaller than 20%, Figure 7G). These counteractive functional changes were associated with a modest deterioration (−9, 7%) of the FMA-UE score.

## Discussion

In this exploratory study, the feasibility and efficacy of multisession bimodal EEG-fMRI NF training for upper limb motor recovery after stroke was tested. The pilot study involved four chronic patients with various degrees of motor impairment and stroke characteristics.

In recent years, few studies have explored the potential of real-time NF for improving motor performances in stroke using different imaging modality such as fMRI (Liew et al., 2016), EEG (Pichiorri et al., 2015; Zich et al., 2017) or functional Near-Infrared Spectroscopy -NIRS (Mihara et al., 2013). A recent systematic review on fMRI NF for motor training in healthy subjects and stroke patients (Wang et al., 2017) indicated that real-time fMRI is effective in promoting self-regulation of targeted areas and has potential to improve motor outcomes. However, the efficacy of fMRI-NF was shown in some but not all studies and depended on function and respective cortical areas engaged. Another factor that severely limits the efficacy of fMRI training is that, due to the cost of MR scanning, in most fMRI-NF studies only short training protocols are usually implemented or tested. This, together with the high complementarity of EEG and fMRI techniques, motivated our effort to integrate these two modalities in view of designing more specific, feasible and effective multi-session training protocols for upper limb motor recovery after stroke.

### Feasibility of Bimodal NF

For the first time in literature, we tested bimodal EEG-fMRI for stroke rehabilitation. Bimodal EEG-fMRI NF poses various challenges: technological (as it requires a complex and high-performance installation), practical (for the relatively long preparation time of the patients, of around 40 min) and mental for the participants (NF is cognitively demanding, particularly in the unfamiliar MRI environment, that the patients usually associate with negative emotions). Results confirmed the feasibility and safety of this protocol on stroke patients with mild to severe hemiparesis: patients managed to upregulate the BOLD activity in the targeted motor areas during NF training. The EEG activity was harder to modulate during bimodal sessions, but all patients successfully upregulated the activity recorded at motor electrodes during unimodal EEG-NF sessions.

In general, a positive response to the training protocol emerged from the questionnaires. Patients were interested and motivated by NF training and the associated challenge and very satisfied with the NF metaphor. They also perceived this type of training as potentially complementary to traditional rehabilitation techniques.

### Multi-target Strategy and Its Relation to Stroke Deficit

One crucial aspect when designing an NF protocol is the choice of target regions. Whether the M1 is activated during motor imagery is still debated (Chiew et al., 2012; Blefari et al., 2015), while SMA seems to be more robustly and easily recruited. This work provides new pieces of evidence that M1 can be activated during motor imagery, especially when the patient is guided to this target through NF.

In this pilot work, we proposed a novel multi-target strategy for a guided rehabilitation of ipsilesional primary motor areas. Such a selective regulation of motor areas is only possible for the fMRI modality, which allows for a more precise spatial identification of activated areas than EEG. The multi-target training was effective in three out of four patients, who improved the activation of ipsilesional M1 in the second training session with respect to the beginning of the protocol. Remarkably, if we consider that the protocol was 1 week long, for two of these patients the improvement of ipsilesional M1 control was associated with an increase of motor performances, as assessed by the FMA-UE score (Figure 3B).

Those two patients exhibited a high degree of symmetry of the CST, and therefore a preserved ipsilesional CST. On the other hand, the patient having a severely impaired CST, with an FA asymmetry index close to the threshold indicating very poor recovery potential, did not exhibit functional improvement.

Patient 04 showed a high integrity of the ipsilesional CST but did not exhibit functional improvement. He presents a large cortical lesion including M1. In addition, the CST (segmented between the posterior limb of the internal capsule and the lower pons) does not seem to reach M1 (Figure 7C). This may be the main reason why the patient fails to activate M1, which is severely damaged while being able to activate SMA (preserved because vascularized by the anterior cerebral artery).

It has been shown that the recovery after partial lesion of M1 at the chronic stage of stroke is associated with reorganization within the surrounding motor cortex (Jaillard et al., 2005). We can hypothesize that, in patients with a large cortical stroke including M1, either recovery of ipsilesional activation would certainly require a much longer NF training, or we should consider changing the cortical target. In this case, contralesional M1 (*via* the cortico-reticulo-propriospinal pathway) would be a relevant alternative target (Bradnam et al., 2013).

These findings highlight the importance of taking into account various factors when designing a clinical protocol. In particular, they confirm the critical role of the preserved neural pathways (the so-called “structural reserve”; Di Pino et al., 2014) in the recovery process and indicate that this is importantly related also to functional brain regulation of the ipsilesional motor cortex, giving useful indications for future studies inclusion criteria.

### Limitations

This is an exploratory work and presents various limitations. The first concerns the challenge of obtaining good quality EEG recordings in the noisy environment of fMRI, which represents the main issue in simultaneous EEG-fMRI recording. Great effort was put to reduce the impedances of the electrodes during patient preparation, strictly following the manufacturer guidelines. However, completely getting rid of BCG and motion artifacts in real-time remains a challenge. BCG artifact correction maybe even more challenging for stroke patients, as they are often affected by atrial fibrillation and therefore have an irregular heart rate, in comparison with the healthy population. The development of more efficient real-time methods to correct these artifacts is the object of current research (Wu et al., 2016) and will considerably improve the quality of bimodal EEG-fMRI NF in the near future. Artifacts and noise are part of the reason why EEG activity regulation during bimodal NF is more challenging if compared to fMRI (Perronnet et al., 2017). Electrophysiological activity may also be intrinsically harder to control than metabolic activity since brain “naturally” regulates and processes feedbacks (i.e., blood pressure or flow) from the vascular system, while there are no equivalent “sensors” for brain electrophysiological activity (Birbaumer et al., 2013).

Another limitation of this study was that we did not control for movements of the affected limb during the motor imagery task by measuring the electromyographic (EMG) signal. This choice was made not to increase the burden and complexity of the simultaneous EEG-fMRI setup, as measuring EMG requires the installation of an additional amplifier at the bottom of the MR bore and, to arrange cables in a straight line, needs custom cable lengths for each individual in order to fulfill the safety regulations and follow the manufacturer guidelines. We have therefore decided to monitor upper limb movements by means of a camera inside the MR bore and repeatedly instructed the patients to remain still during NF training.

Here we present results from a pilot study and further research is required to validate its findings and assess the efficacy of bimodal EEG-fMRI for stroke rehabilitation. The lack of blinded assessment and the absence of a control group (for instance a group receiving sham NF or a treatment-as-usual group) does not allow to rigorously assess if patients upregulated their brain activity by means of the NF training neither to determine if the observed clinical effects are a result of the NF intervention, as the observed improvement may be related to other uncontrolled factors (Sorger et al., 2018).

This is an exploratory study whose main aims were to test the feasibility of the bimodal EEG-fMRI NF training in stroke patients and identify critical aspects for the design of a randomized controlled study. Our preliminary results are however encouraging and indicate that motor improvements were obtained after a relatively short training duration (1 week) in two out of four chronic patients at more than 1 year from the stroke episode, where spontaneous recovery has stopped. They also support the hypothesis that in these patients NF may trigger functional reorganization of the affected motor areas by exploiting the residual brain plasticity. Finally, this pilot study was useful to identify crucial aspects and inclusion criteria for the design of a larger randomized controlled trial on chronic stroke patients.

## Conclusion

In this exploratory study, the feasibility and efficacy of bimodal EEG-fMRI NF training for upper limb motor recovery was tested in four chronic stroke patients. Preliminary results indicate that success in upregulating the activity of target motor areas depends on the type and severity of the stroke damage and stress the importance of taking into account the variability of the stroke patients’ population when designing a clinical protocol. These findings give useful indications for the design of future clinical studies with NF.

## Data Availability Statement

The datasets generated for this study are available on request to the corresponding author.

## Ethics Statement

The studies involving human participants were reviewed and approved by Comité de Protection des Personnes de Poitiers Ouest III. The patients/participants provided their written informed consent to participate in this study.

## Author Contributions

All authors contributed to the study design, read and commented the manuscript. GL performed data collection, analysis and wrote the first draft of the manuscript. SB performed patients’ enrollment, screening and motor assessment, contributed to the discussion of results and revised the manuscript. MF contributed to data collection and revised the manuscript. EB provided assistance in designing the study and in data collection and revised the manuscript. AL, IB, and CB provided feedback on data analysis and interpretation, discussed the results and revised the manuscript.

## Conflict of Interest

The authors declare that the research was conducted in the absence of any commercial or financial relationships that could be construed as a potential conflict of interest.

## Footnotes

^1^http://psychtoolbox.org/

^2^https://www.mensia.com/

^3^http://med.inria.fr/

^4^http://www.fieldtriptoolbox.org
